# Tolosa Hunt Syndrome: MRI Findings

**DOI:** 10.7759/cureus.46635

**Published:** 2023-10-07

**Authors:** Jerome A Ramirez, Estefania Ramirez Marquez, Gerardo Torres, Claudia Muns Aponte, Eduardo J Labat

**Affiliations:** 1 Radiology, University of Puerto Rico School of Medicine, Medical Sciences Campus, San Juan, PRI; 2 Ophthalmology, University of Puerto Rico School of Medicine, Medical Sciences Campus, San Juan, PRI; 3 Neuroradiology, University of Puerto Rico, Medical Sciences Campus, San Juan, PRI

**Keywords:** radiology, mri, neuroimaging, cavernous sinus, tolosa hunt syndrome

## Abstract

Tolosa-Hunt syndrome (THS) is an idiopathic inflammatory condition involving the cavernous sinus and orbital apex with an incidence of 1 case per million per year. We report on a case of a 70-year-old male with atypical MRI findings, vision loss, and painless ophthalmoplegia.  Ophthalmic evaluation revealed his best-corrected visual acuity was 20/40 in the right eye and counting fingers at a 0.5-foot distance in the left eye. External examination of the left eye revealed limited ocular movement, proptosis, and a positive relative afferent pupillary defect. Complete blood count, inflammatory markers, and full biochemistry tests, including thyroid and liver function tests, were within the normal range. A magnetic resonance imaging of the orbits with and without contrast demonstrated a homogenously enhancing lesion at the posterior intraconal compartment of the left orbit, extending to the orbital apex with the involvement of the adjacent extraocular muscles. The patient was started on intravenous methylprednisolone 60 mg daily and later discharged on prednisone 5 mg daily with partial symptom improvement on follow-up. Resection and biopsy revealed a soft tissue lesion with mixed inflammatory infiltrate. The clinical, pathological, and imaging findings favored the diagnosis of THS.

## Introduction

Tolosa-Hunt syndrome (THS) is an idiopathic inflammatory condition involving the cavernous sinus and orbital apex [1-8]. The inflammation causes extrinsic compression of the neurovascular structures that cross the cavernous sinus [3,6,9,10]. This disorder is caused by the infiltration of lymphocytes and plasma cells, along with the thickening of dura mater within the cavernous sinus [11]. Patients typically present orbital pain accompanied by paresis of the III, IV, or VI cranial nerves (CN), commonly leading to diplopia [4,11]. The incidence of THS is 1 case per million per year with an average age of presentation of 40 years [4,5,11]. Recent viral-mediated infections have been proposed as an important risk factor for this disease.

In the time before the ready availability of imaging technologies, THS was largely a diagnosis of exclusion, based on clinical presentation and response to steroid therapy [1]. Diagnostic criteria now include the presence of unilateral headache, granulomatous inflammation of the cavernous sinus, superior orbital fissure or orbit, supported by Magnetic Resonance Imaging (MRI) or biopsy, and paresis of ipsilateral CN III, IV, or VI [12]. The preferred imaging modality for evaluating THS is MRI, primarily because it helps in both confirming the presence of granulomatous inflammation and ruling out alternative conditions affecting the cavernous sinus, including lymphoma [1-3,6,9-11].

We present herein the atypical MRI findings of a 70-year-old male experiencing vision loss and painful ophthalmoplegia 14 days after cataract surgery.

## Case presentation

A 70-year-old male presented to our emergency department with vision loss and limited eye movements in the right eye, persisting for five days. The patient's medical history was notable for hypertension and cataract surgery performed 14 days prior. His review of systems indicated left-sided headaches and facial numbness. His social and family history were otherwise unremarkable.

Upon ophthalmic evaluation, his best-corrected visual acuity was 20/40 in the right eye and counting fingers at a 0.5-foot distance in the left eye. External examination showed that extraocular movements were within normal limits for the right eye. The left eye exhibited limited ocular movement, proptosis, and a positive relative afferent pupillary defect. Complete blood count, inflammatory markers, and comprehensive biochemistry tests, including thyroid and liver function tests, were all within normal limits.

**Figure 1:**
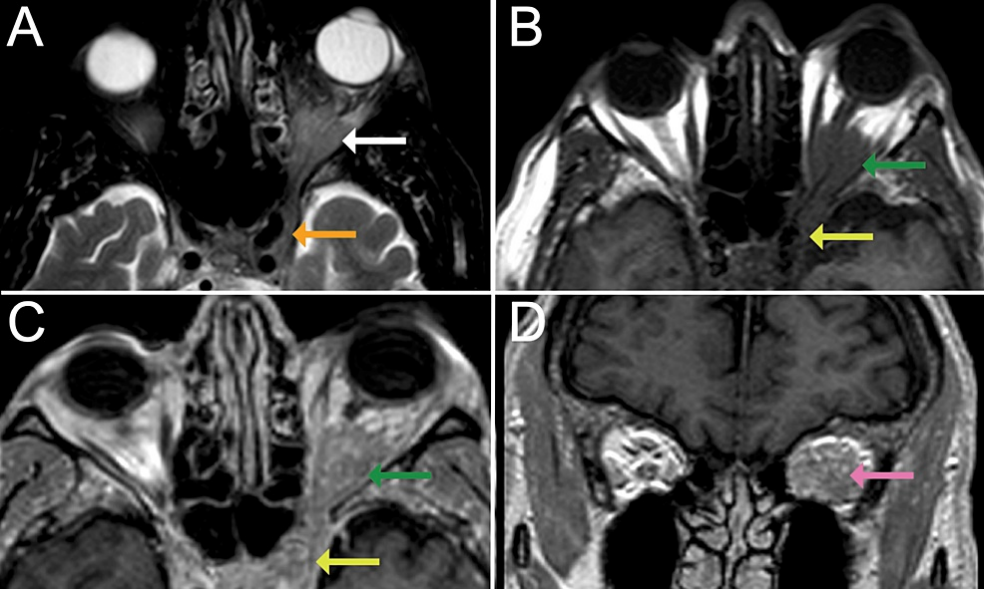
Brain MRI images. T2 weighted Brain MRI axial sequence demonstrating a T2 hypointense lesion (white arrow) centered in the mid- to posterior intraconal compartment of the left orbit involving the intraocular muscles near the region of the orbital apex. (A) The lesion is seen compressing the optic nerve sheath displacing it superomedially. Similar signal intensity is observed within the left cavernous sinus consistent with sinus invasion (orange arrow). Axial (B) pre- and (C) post-contrast T1 weighted sequences demonstrating homogeneous enhancement of the orbital lesion (green arrow) with associated proptosis and extension to the cavernous sinus (yellow arrow). (D) Coronal post-contrast T1 weighted MR sequence demonstrating homogeneous enhancement of the orbital lesion (pink arrow).

An MRI scan of the orbits, both with and without contrast, was performed using a Philips Achieva model scanner with a 1.5-tesla magnet. The MRI revealed a homogeneously enhancing lesion in the posterior intraconal compartment of the left orbit, extending to the orbital apex and involving the adjacent extraocular muscles (Figure 1). Given the clinical findings described above, these results were most consistent with idiopathic orbital inflammatory pseudotumor, falling within the spectrum of THS, versus lymphoma.

During admission, the patient was treated with intravenous methylprednisolone 60 mg and then discharged on prednisone 5 mg daily per oral with partial symptom improvement on follow-up. Resection and biopsy of the orbital apex soft tissue was subsequently performed demonstrating a soft tissue lesion with mixed inflammatory infiltrate. These findings favored THS rather than a neoplastic lesion such as lymphoma.

## Discussion

Reports of THS are limited and display a wide array of clinical presentations and associated findings [1-11]. THS has been previously reported in conjunction with autoimmune inflammatory disorders such as systemic lupus erythematosus, sarcoidosis, and Wegener's granulomatosis [1,4,5,7,8,11]. To the best of our knowledge, this is the first case of THS reported to occur following cataract surgery, specifically 14 days after the procedure. While this could be merely coincidental, we must consider the possibility that the surgery either triggered or exacerbated an existing inflammatory state.

The existing literature on THS indicates that it is a granulomatous inflammatory condition affecting the cavernous sinus, which may extend to the superior orbital fissure, orbital apex, or optic nerve [5,11]. This granulomatous inflammation often involves cranial nerves III, IV, or VI [11]. Although the etiology of this inflammatory condition remains idiopathic, recent viral-mediated infections have been suggested as a significant risk factor [11].

The International Headache Society diagnostic criteria classify THS under painful cranial neuropathies and other facial pains [4,5]. According to the criteria, a diagnosis of THS requires a unilateral headache and evidence of causation, either by the headache being preceded by paresis of cranial nerves III, IV, and VI by up to two weeks or developing concurrently. Additionally, the headache should be localized around the ipsilateral brow and eye, and there should be granulomatous inflammation of the affected sites, as demonstrated by MRI or biopsy, with paresis of one or more of the ipsilateral cranial nerves III, IV, and/or VI [1-12].

Specific clinical diagnostic criteria are delineated; however, literature on the radiological aspect of the diagnosis remains ambiguous [2,3,6,9,10]. This may be due to the rarity of the disease as well as the difficulty in creating detailed images of the cavernous sinus [2,3,9,10,12]. Currently, MRI is the imaging modality of choice for evaluating THS, and to exclude other lesions that can involve the cavernous sinus such as lymphoma [1,4,7]. Suggestive findings of THS in MRI include enlargement and dural margin convexity, with or without T1-isointense with gray matter abnormal tissue, and isointense to hypointense on T2 sequences [2,3,6,9,10,12]. A limitation, however, is that MRI detects any abnormal tissue, not only granulomatous inflammation [2,3,6,9,10].

MRI changes pre- and post-steroid therapy may confirm the diagnosis of THS; however, radiological resolution takes months to appear [1]. Authors have also reported a small percentage of patients with normal MRI imaging at presentation [1]. When steroid response is considered a criterion, studies have found that between 18.18% to 57% of patients with THS had normal imaging [1,2]. This variation could be due to the images being taken at different stages of the disease [1-7,9,10]. Therefore, repeating an MRI when following up on patients may reveal lesions in the cavernous sinus [1-7,9,10].

MRI is a very useful tool as it may contribute valuable data about a patient’s disease in a noninvasive manner [2,3,6,9,10]. Tissue biopsy may be used to directly demonstrate granulomatous inflammation; however, this may prove to be a challenging procedure and entail increased risks for the patient [11]. Although THS generally has a positive prognosis, correct identification and prompt treatment of this disease are vital, as complications such as aberrant nerve regeneration and a relapsing-remitting course may occur [4,11]. This text should serve to highlight the importance of continuing to improve the characterization of neuroimaging studies of THS, as well as to describe this patient’s unique presentation following cataract surgery. The possibility that the surgery triggered or exacerbated an existing inflammatory state should be considered; however, further studies are needed to elucidate this potential relationship.

## Conclusions

Tolosa-Hunt syndrome is an extremely rare inflammatory condition with a variable clinical presentation and response to treatment. It is primarily a diagnosis of exclusion. Patients often present with painful ophthalmoplegia that improves with steroid therapy. While specific imaging criteria do not exist, MRI can serve as a valuable tool in supporting the diagnosis. The radiologist must recognize MRI findings suggestive of THS, as well as differentiate other causes of painful ophthalmoplegia, to establish appropriate management and follow-up. Although the potential link between cataract surgery and the triggering or exacerbation of inflammation is noteworthy, further research is needed to clarify this possible association.
